# Prevalence and severity of long‐term physical, emotional, and cognitive fatigue across 15 different cancer entities

**DOI:** 10.1002/cam4.3413

**Published:** 2020-09-07

**Authors:** Martina E. Schmidt, Silke Hermann, Volker Arndt, Karen Steindorf

**Affiliations:** ^1^ Division of Physical Activity, Prevention and Cancer National Center for Tumor Diseases (NCT) and German Cancer Research Center (DKFZ) Heidelberg Germany; ^2^ Epidemiological Cancer Registry Baden‐Württemberg German Cancer Research Center (DKFZ) Heidelberg Germany; ^3^ Division of Clinical Epidemiology and Aging Research German Cancer Research Center (DKFZ) Heidelberg Germany

**Keywords:** breast cancer, cancer survivorship, cancer‐related fatigue, gastrointestinal cancer, prevalence, quality of life

## Abstract

**Background:**

Fatigue prevalence and severity have been assessed in a variety of studies, yet, not in a standardized way, and predominantly in breast cancer patients. Systematic, comparative investigations across a broad range of cancer entities are lacking.

**Methods:**

The FiX study systematically enrolled 2244 cancer patients across 15 entities approximately 2 years after diagnosis. Fatigue was assessed with the multidimensional EORTC QLQ‐FA12 questionnaire. Physical, emotional, cognitive, and total fatigue were compared across entities and with normative values of the general population. Differences in patients' characteristics and cancer therapy between entities were taken into account using analyses of covariance models.

**Results:**

Across all entities, mean physical fatigue levels were significantly higher than age‐ and sex‐matched means of the general population for all cancer entities (all Bonferroni‐Holm adjusted *P* < .01). For most entities also emotional and cognitive fatigue levels were significantly higher than normative values. Age‐ and sex‐standardized physical fatigue prevalence ranged from 31.8% among prostate to 51.7% among liver cancer patients. Differences between entities could not be fully explained by sex, age, BMI, or cancer therapy. Adjusted for these factors, mean physical fatigue was higher for stomach (*P* = .0004), lung (*P* = .034), kidney (*P* = .0011), pancreas (*P* = .081), and endometrium (*P* = .022) compared to breast cancer patients. Adjusted means of emotional fatigue were also lowest in breast cancer patients and significantly higher in stomach (*P* = .0047), bladder (*P* = .0036), and rectal (*P* = .0020) cancer patients.

**Conclusions:**

Physical, emotional, and cognitive fatigue is prevalent in all 15 investigated cancer entities even 2 years after diagnosis. Fatigue in breast cancer patients, the so‐far most studied group, is in the lowest range among all entities, suggesting that the extent of fatigue is still insufficiently determined. Entity‐specific problems might need to be considered in the treatment of fatigue.

## Graphical abstract

The FiX study compared fatigue in 2244 cancer patients across 15 tumor entities. Fatigue was prevalent even 2 years after diagnosis across all entities, but with different manifestation. We identified entity‐specific issues to be considered in the treatment of fatigue.

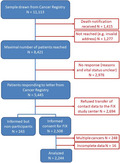

## 1 INTRODUCTION

Cancer‐related fatigue is a frequent and burdensome symptom that has been observed across different cancer entities and therapies.^1^ Fatigue can manifest in various dimensions such as physical, emotional and cognitive exhaustion. It varies in intensity as well as in the temporal course.^2^ Reported prevalence of fatigue during cancer treatment ranged from 25% to 99%, and in one quarter to one third of cancer survivors fatigue persisted for up to 10 years after end of therapy.^1^, ^3^ So‐far, breast cancer was the most frequent entity included in studies on fatigue,^4^, ^5^, ^6^ but there is ample evidence that other patient groups may also be in need for fatigue management. To better determine those groups, comparable information on fatigue prevalence and severity are needed. Many studies have presented fatigue prevalence within their study populations. However, comparability of these data is hindered by several aspects: (1) Heterogeneity of assessment: Clear objective measures for fatigue do not exist. Instead, a variety of questionnaires have been typically used to assess fatigue. (2) Cut‐off points: Fatigue is not a dichotomous “yes/no‐variable” but exerts its intensity on a continuous scale. Thus, fatigue prevalence depends on the respective selected cut‐off. (3) Differences in study populations: Reporting of fatigue depends on the time point of assessment in relation to cancer therapy, the type of treatment, and individual patient characteristics such as age, sex, physical, or psychological condition.

To the best of our knowledge there is only one publication that reported uniformly determined fatigue prevalence for more than four cancer entities.^7^ This study enrolled 1494 patients between 2002 and 2004, from only one hospital in Germany. Other studies exist that compared only three or four cancer types.^8^, ^9^, ^10^

Therefore, our FiX study aimed to systematically assess and compare prevalence of fatigue across the 15 most frequent cancer entities. Hereby, different time points and dimensions of fatigue were considered. Moreover fatigue scores were compared to normative values of the general German population.

## 2 METHODS

### 2.1 Study population

**Figure 1:**
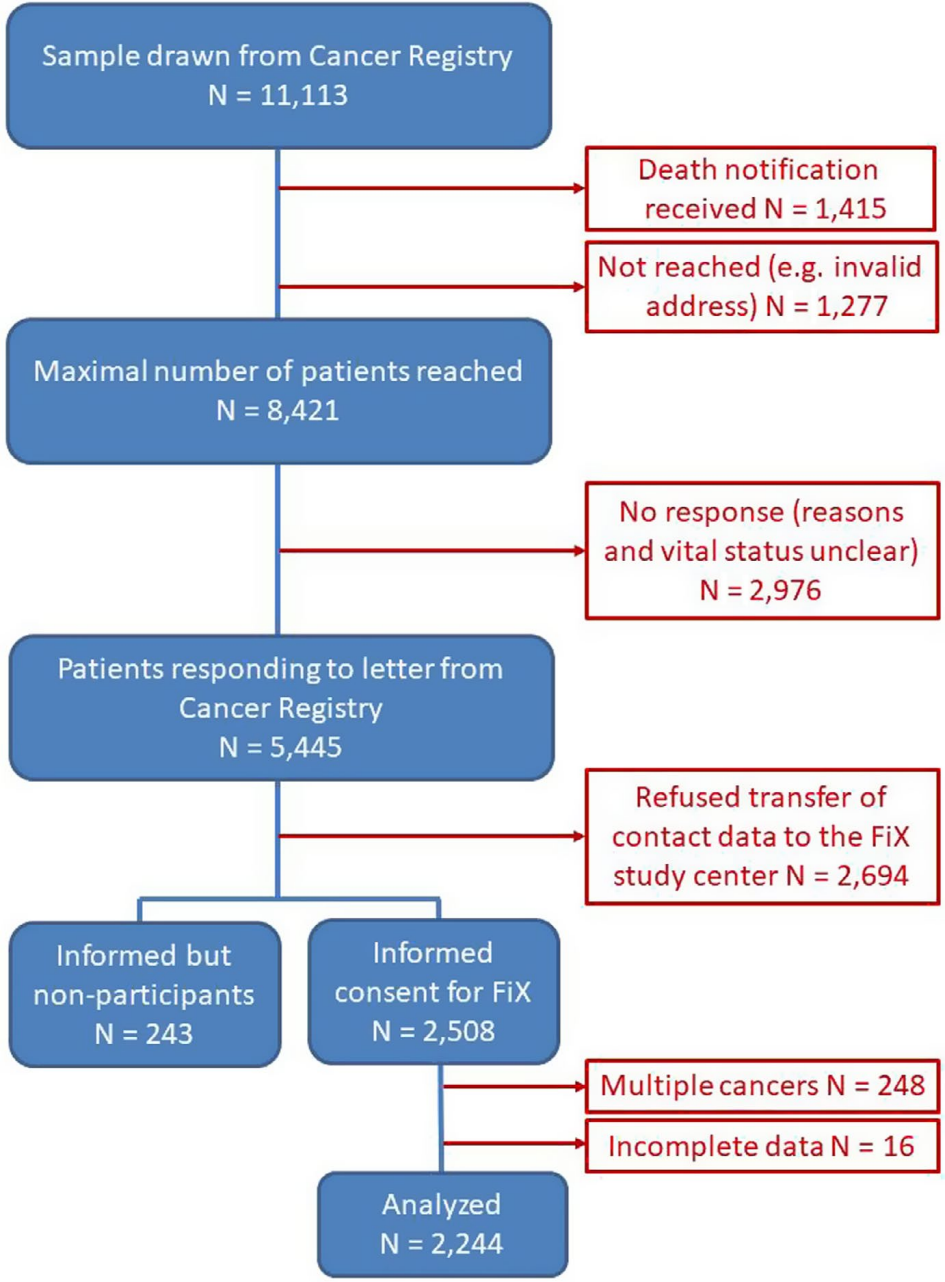
CONSORT diagram

The FiX study (**F**atigue **i**n Germany ‐ E**x**amination of prevalence, severity, and state of screening and treatment) recruited patients between March 2018 and May 2019 via the Epidemiological Cancer Registry of Baden‐Württemberg, Germany. Patients aged 18+ years were eligible if diagnosed with a primary tumor of following entities: stomach (C16, D00.2), colon (C18, D01.0), rectum (C19‐20/D01.1‐1.2), liver (C22/D01.5), pancreas (C25/D01.7), lung (C33‐34/D02.1‐2.2), malignant melanoma (C43/D03), breast (C50/D05), endometrium (C54.1/D07.0), ovaries/cervix (C56/C53/D06), prostate (C61), kidney (C64), bladder (C67/D09.0), non‐Hodgkin lymphoma (C82‐88), leukemia (C91‐C95). To assess fatigue in the longer run, patients were recruited about 2 years after diagnosis. A population‐based sample of the cancer registry stratified by entity was drawn in two batches. To reach proximally balanced numbers across entities, sampling of the second batch was based on the return from the first batch. As in the first batch many liver, lung, and pancreas cancer patients had died (death notices to the cancer registry were still pending at time of sampling), these entities were excluded from second sampling to reduce the emotional burden for the relatives caused by receiving letters for the deceased family member. Following data protection laws, the cancer registry sent letters to the sampled patients, asking for permission to transfer contact data to the study center. Of 11113 sampled patients, 1277 could not be reached (eg invalid/unknown address) and in 1415 cases it turned out that the patient had already died (Figure 1). Thus, 8421 patients may have been reached by the cancer registry, however, from 2976 of these patients no feedback was received whereas 2694 actively refused the transfer of contact data to the study center. Of the remaining patients, 2508 gave informed consent to participate (29.8% of the potentially reached patients). We excluded 248 patients from the prevalence analysis, due to primary cancers in multiple entities. Further 16 patients did not complete the fatigue questionnaires, leaving 2244 patients included for the final analyses.

### 2.2 Data collection

Fatigue was self‐reported by the patients using the EORTC QLQ‐FA12, a 12‐item, multidimensional questionnaire developed for cancer patients.^11^ It covers the physical, emotional, and cognitive dimensions of fatigue and total fatigue, has good test‐retest reliability and internal consistency and been proved to identify clinically significant changes in fatigue in the course of cancer treatment.^11^, ^12^ Body‐mass index (BMI) was calculated from self‐reported weight and height. Cancer therapy was self‐reported and supplemented by cancer registry data. Age, sex, and cancer entity was derived from registry data. Normative values of the EORTC QLQ‐FA12 are available from 2411 individuals, representatively selected from the German general population, stratified by sex and age.^13^

### 2.3 Statistical methods

Physical, emotional, cognitive, and total fatigue scores were derived from the EORTC QLQ‐FA12 and transformed to the range 0‐100 according to the EORTC scoring manual. Higher numbers indicate higher degrees of fatigue. Fatigue scores were compared between entities with three approaches. First, raw physical, emotional, cognitive, and total fatigue scores were presented in Box‐Whisker‐plots and compared been entities using Kruskal‐Wallis tests. Additionally, the raw fatigue scores were compared with the age‐ and sex‐matched mean scores of the general German population^13^ using paired *t* tests. We adjusted for multiple testing across all entities using the Bonferroni‐Holm method. Secondly, age‐ and sex‐standardized prevalences were calculated. The cut‐off for abnormal high EORTC QLQ‐FA12 scores was defined by the age‐ and sex‐specific 75th percentile from the general population^13^ according to Singer et al^7^ Hence, we chose it to enable comparisons of our data with those previous findings. Thirdly, fatigue differences between entities adjusted for age (linear and quadratic term), sex (male, female), BMI (<18.5, 18.5‐<25, 25‐<30, 30‐<35, ≥35), surgery, chemo‐, radio‐, targeted, and endocrine therapy (never, within the last 4 weeks, more than 4 weeks ago) were explored using analyses of covariance (ANCOVA). Adjusted means enable comparison of fatigue across cancer entities irrespective of different patients' characteristics. The ANCOVA was generally explorative in nature, however, we in addition considered ANCOVA results using adjustment for multiple testing according to Dunnett‐Hsu. Since the number of missing values is below 4%, ANCOVA models were based on complete cases without missing imputations. All tests were two‐sided using 5% significance level, and SAS version 9.4.

## 3 RESULTS

The mean age of the study population was 65.5 years, gender was evenly distributed, and the mean BMI of 26.9 is similar to the mean BMI of the general population, and gender was evenly distributed (Table 1). Although we had aimed for about N = 200 patients per entity, the final numbers differed due to response rate and rate of the already deceased. Breast cancer was the most frequent entity (N = 230, response rate 40%, deceased 4%), whereas only 125 stomach cancer patients could be included (response rate 25%, deceased 30%).

**Table 1:** Characteristics of study population (n = 2244)

**Characteristics**	
Age at enrolment, mean (SD)	65.6 (11.9)
<40 years	51 (2.3%)
40‐<50 years	154 (6.9%)
50‐<60 years	519 (23.1%)
60‐<70 years	662 (29.5%)
70‐<80 years	620 (27.6%)
≥80 years	238 (10.6%)
Sex	
Male	1131 (50.4%)
Female	1105 (49.2%)
Missing	8 (0.4%)
BMI (kg/m^2^), mean (SD)	26.9 (5.5)
<18.5	38 (1.7%)
18.5‐25	871 (38.8%)
25‐30	798 (35.6%)
30‐35	337 (15.0%)
≥35	148 (6.6%)
Missing	52 (2.3%)
Years since diagnosis, mean (SD)	1.8 (0.4)
>1‐1.5 years	458 (20.4%)
>1.5‐2 years	1027 (45.8%)
>2‐2.5 years	656 (29.2%)
>2.5‐3 years	81 (3.6%)
>3‐3.5 years	9 (0.4%)
>3.5‐5 years	13 (0.6%)
Entity of cancer disease	
Breast	230 (10.2%)
Prostate	220 (9.8%)
Kidney	206 (9.2%)
Non‐Hodgkin lymphoma	204 (9.1%)
Rectum	191 (8.5%)
Colon	185 (8.2%)
Endometrium	174 (7.8%)
Malignant melanoma	166 (7.4%)
Leukemia	158 (7.0%)
Ovaries/Cervix	147 (6.6%)
Bladder	139 (6.2%)
Stomach	125 (5.6%)
Lung	37 (1.6%)
Pancreas	33 (1.5%)
Liver	29 (1.3%)
**Cancer treatment**	
Chemotherapy	
Never	1312 (58.5%)
In the past	798 (35.6%)
Recent/current	115 (5.1%)
Missing	19 (0.8%)
Radiotherapy	
Never	1620 (72.2%)
In the past	587 (26.2%)
Recent/current	22 (1.0%)
Missing	15 (0.7%)
Targeted therapy	
Never	1810 (80.7%)
In the past	304 (13.5%)
Recent/current	113 (5.0%)
Missing	17 (0.8%)
Endocrine therapy	
Never	1871 (83.4%)
In the past	216 (9.6%)
Recent/current	140 (6.2%)
Missing	17 (0.8%)
Surgery	
Never	379 (16.9%)
In the past	1796 (80.0%)
Recent/current	36 (1.6%)
Missing	33 (1.5%)

**Figure 2:**
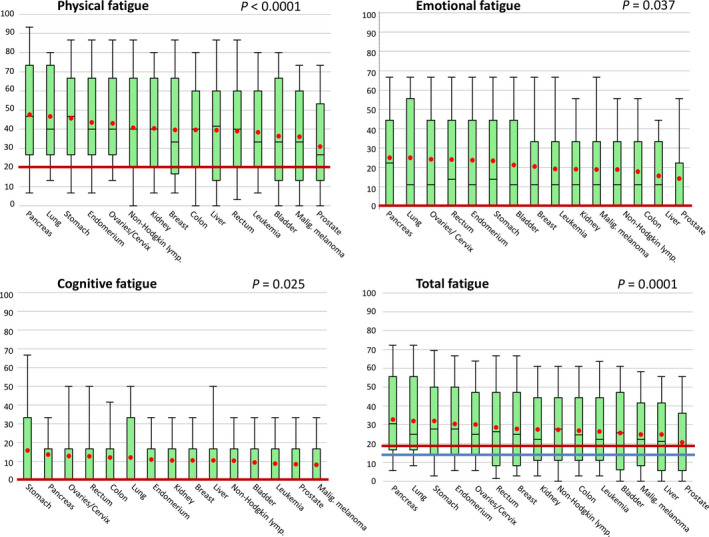
Box‐Whisker plots of raw physical, emotional, cognitive, and total fatigue scores. Boxes represents 25th to 75th percentiles with middle line in box at median, Whisker ends at 10th and 90th percentiles; red dots represent mean values. The median normative fatigue values of the German population of age 60+ years are presented by a blue line for males, and red line for females or both sexes, if median identical

Figure 2 presents the distributions of the raw fatigue scores across the different entities. Physical (*P* = .0001), emotional (*P* = .0059), cognitive (*P* = .036), and total (*P* = .0002) fatigue scores differed significantly between entities. The median (Q1, Q3) physical fatigue ranged from 26.7 (13.3, 53.3) in prostate cancer patients to 46.7 (26.7, 66.7) and 46.7 (26.7, 73.3) in stomach and pancreas cancer patients, respectively. Emotional and total fatigue levels were also lowest among prostate cancer patients. Mean physical fatigue levels were significantly higher than age‐ and sex‐matched means of the general population for all cancer entities (paired *t* test, all Bonferroni‐Holm adjusted *P* < .01). Mean emotional fatigue levels were significantly higher for all entities except liver cancer (paired *t* test, Bonferroni‐Holm adjusted *P* = .16), and mean cognitive fatigue for all entities except pancreas (Bonferroni‐Holm adjusted *P* = .059), leukemia (*P* = .059), liver (*P* = .18), lung (*P* = .18), and malignant melanoma (*P* = .18). The cognitive fatigue score reported in the general population is predominantly zero, indicating no cognitive exhaustion (80% of male and 78% of female of age 60+). Cognitive fatigue was also rated as zero for over 50% of cancer patients. Since the cognitive fatigue score (based on only 2 items) is not very distinctive, no further analyses are presented for the cognitive fatigue dimension. The distributions of physical, emotional as well as total fatigue are presented for sex and age subgroups in Tables [Link], [Link], [Link]. The mean fatigue values and differences from age‐ and sex‐matched normative values are presented by entity in Table S4.

Age‐ and sex‐standardized fatigue prevalence (Table 2) differs across tumor entities (physical fatigue *P*_Chi_ = 0.092, emotional *P*_Chi_ = 0.0038, total fatigue *P*_Chi_ = 0.089). For physical fatigue, prevalence ranged from 31.8% (95% CI: 25.6%‐38.0%) among prostate cancer patients to 51.7% (33.5%‐69.9%) among liver cancer patients.

**Table 2:** Age‐ and sex‐standardized prevalence^a^ of different fatigue dimensions by tumor entity

Entity^b^	N	Prevalence (%) with 95% confidence interval		
		Physical fatigue	Emotional fatigue	Total fatigue
Liver	29	51.7 (33.5, 69.9)	34.5 (17.2, 51.8)	48.3 (30.1, 66.5)
Stomach	121	51.6 (42.8, 60.5)	45.5 (36.6, 54.3)	50.0 (41.1, 58.9)
Lung	35	51.4 (34.9, 68.0)	40.0 (23.8, 56.2)	45.7 (29.2, 62.2)
Pancreas	33	48.5 (31.4, 65.5)	48.5 (31.4, 65.5)	54.5 (37.6, 71.5)
Kidney	205	45.4 (38.6, 52.2)	38.0 (31.4, 44.7)	42.9 (36.2, 49.7)
Non‐Hodgkin lymph.	203	45.3 (38.5, 52.2)	41.9 (35.1, 48.7)	45.3 (38.5, 52.2)
Rectum	188	45.3 (38.2, 52.3)	45.3 (38.2, 52.3)	46.8 (39.7, 53.9)
Colon	182	44.0 (36.7, 51.2)	34.1 (27.2, 41.0)	45.1 (37.8, 52.3)
Ovaries/Cervix	145	42.8 (34.7, 50.8)	35.2 (27.4, 42.9)	44.8 (36.7, 52.9)
Malignant melanoma	162	42.0 (34.4, 49.6)	33.3 (26.1, 40.6)	40.7 (33.2, 48.3)
Endometrium	172	41.9 (34.5, 49.2)	36.3 (29.1, 43.5)	47.1 (39.6, 54.6)
Leukemia	157	39.9 (32.2, 47.5)	36.7 (29.2, 44.2)	44.9 (37.2, 52.7)
Breast	228	39.9 (33.6, 46.3)	31.0 (25.0, 37.0)	41.0 (34.7, 47.4)
Bladder	138	38.4 (30.3, 46.5)	49.3 (40.9, 57.6)	44.9 (36.6, 53.2)
Prostate	217	31.8 (25.6, 38.0)	29.7 (23.6, 35.7)	31.1 (24.9, 37.2)

^a^ Patients with scores above the age‐ and sex‐specific 75% percentile of the general German population are considered fatigued.

^b^ Sorted by prevalence of physical fatigue.

Multivariate, adjusted ANCOVA analyses are summarized in Table 3. Breast cancer patients showed the lowest adjusted mean physical fatigue values, and compared to them the levels in stomach (*P* = .0004), lung (*P* = .034), kidney (*P* = .0011), pancreas (*P* = .081), and endometrium (*P* = .022) were markedly higher. When adjusting for multiple testing, the differences between stomach and kidney cancer compared to breast cancer still remained statistically significant. Adjusted means of emotional as well as total fatigue were also lowest for breast cancer and highest for stomach cancer. Bladder and rectum cancer patients reported significantly higher emotional fatigue, and renal cancer patients significantly higher total fatigue than breast cancer patients.

**Table 3:** Physical, emotional, and total fatigue adjusted by age, sex, BMI and cancer treatment

Entity^a^	Physical fatigue		Emotional fatigue		Total fatigue	
	Adjusted means (95% CI)	*P* (difference to breast cancer)	Adjusted means (95% CI)	*P* (difference to breast cancer)	Adjusted means (95% CI)	*P* (difference to breast cancer)
Stomach	69.4 (61.6, 77.2)	**0.0004** *	42.4 (35.0, 49.9)	**0.0047** *	50.0 (43.5, 56.4)	**0.0013** *
Lung	66.9 (55.8, 78.0)	**0.034**	39.5 (28.9, 50.1)	0.13	46.9 (37.7, 56.0)	0.10
Kidney	66.6 (59.4, 73.8)	**0.0011** *	40.7 (33.9, 47.6)	**0.0069**	47.8 (41.9, 53.7)	**0.0038** *
Pancreas	65.0 (53.5, 76.5)	0.081	37.1 (26.1, 48.1)	0.31	45.9 (36.4, 55.4)	0.16
Endometrium	62.7 (55.3, 70.1)	**0.022**	37.7 (30.6, 44.8)	0.056	44.7 (38.6, 50.8)	0.052
Liver	61.9 (50.4, 73.4)	0.23	33.3 (22.3, 44.3)	0.72	41.8 (32.3, 51.3)	0.58
Leukemia	61.7 (53.9, 69.4)	0.10	39.1 (31.7, 46.6)	0.056	44.5 (38.1, 50.9)	0.13
Ovaries/Cervix	61.5 (53.8, 69.1)	0.079	38.2 (30.9, 45.5)	0.063	43.5 (37.2, 49.8)	0.17
Colon	61.5 (54.2, 68.8)	0.07	36.6 (29.6, 43.6)	0.14	43.9 (37.8, 49.9)	0.12
Bladder	60.8 (53.2, 68.4)	0.12	42.4 (35.1, 49.6)	**0.0036** *	44.8 (38.5, 51.1)	0.082
Rectum	60.7 (53.6, 67.7)	0.097	41.9 (35.1, 48.6)	**0.0020** *	45.4 (39.5, 51.2)	**0.034**
Malignant melanoma	60.4 (53.1, 67.8)	0.12	38.4 (31.3, 45.4)	0.046	43.1 (37.0, 49.2)	0.19
Non‐Hodgkin lymphoma	59.2 (52.5, 66.0)	0.22	35.5 (29.1, 41.9)	0.23	41.8 (36.2, 47.3)	0.38
Prostate	55.8 (48.9, 62.8)	0.76	36.2 (29.6, 42.8)	0.14	40.6 (34.9, 46.3)	0.59
Breast	54.8 (47.8, 61.7)	Ref.	31.3 (24.7, 37.9)	Ref.	39.1 (33.4, 44.8)	Ref.

Significant differences to breast cancer (*P* < .05) are marked as bold values.

Abbreviations: BMI, Body mass index; CI, Confidence interval.

^a^ Ordered by adjusted means of physical fatigue.

* Significant (*P* < .05) after Dunnett‐Hsu adjustment for multiple testing.

## 4 DISCUSSION

The Fix study systematically assessed and compared fatigue prevalence and severity for 15 cancer entities among 2,244 cancer patients, differentiating by dimension of fatigue. For all entities, physical fatigue scores in cancer patients 2 years after diagnosis were significantly higher than in the general German population, and for the vast majority of entities significant differences were also seen for emotional and cognitive fatigue. Yet, our results indicated clear differences between entities. Physical fatigue prevalence ranged from 31.8% among prostate to 51.7% among liver cancer patients. Differences between entities were not fully explained by sex, age, BMI, or type and timing of cancer therapy, as after adjusting for those factors fatigue still differed significantly among tumor entities, with highest adjusted mean levels of physical, emotional, and total fatigue in stomach and lowest levels in breast cancer patients.

The considerable observed physical fatigue prevalence of 40% among breast cancer patients about 2 years after diagnosis indicates that an effective fatigue management and treatment is not yet established. The prevalence is in similar magnitude as published from another study with comparable prevalence calculation (36%).^7^ Compared to breast cancer, fatigue values were higher in most other investigated entities. Thus, improvements in fatigue management might be needed even more urgently for patients with other types of cancer. Given the fact that most research on cancer‐related fatigue so far has been conducted with breast cancer patients, the scope of the problem and potential therapies likely have not yet been fully explored. Likewise, randomized controlled trials considering the so‐far promising treatment approaches for fatigue, that is, physical exercise, yoga or other mind‐body exercise, and psychosocial interventions such as cognitive behavioral therapy or mindfulness‐based stress reduction, have predominantly included breast cancer patients.^14^, ^15^, ^16^, ^17^, ^18^, ^19^ Overall, there is convincing evidence that physical activity and exercise is beneficial for breast cancer patients in the adjuvant setting. Survivors of (non‐metastasized) breast cancer often have less functional restrictions than patients with other tumor entities, are known to frequently use rehabilitation and continue to engage in physical activities. Thus, future research should focus on patients with cancers of other entities where evidence on fatigue therapies is weak.

One reason that adjusted physical fatigue levels were significantly higher among patients with stomach, lung, pancreas, and kidney cancer compared to breast cancer might be the specific course of disease: Since we collected data concerning fatigue approximately 2 years after diagnosis, for these cancers with poorer prognosis it is conceivable that patients are more likely to be in worse condition due to disease progression and might have already received several lines of therapy until this time point. This in return has been shown to be associated with increased fatigue levels.^20^ However, also entity specific factors may need to be taken into consideration, as discussed in the following.

Stomach cancer patients showed high physical and emotional fatigue about 2 years after diagnosis. A study investigating 374 disease‐free stomach cancer patients also found a high fatigue prevalence of 51.3% (determined as global BFI score of ≥4).^21^ Stomach cancer patient may suffer from postgastrectomy syndrome, which can result in malnutrition, loss of skeletal muscle mass, and anemia, and thus contribute to physical fatigue. Similar problems can also arise after pancreatic cancer, and likewise might contribute to the reported high physical fatigue levels.

Renal and lung cancer was associated with high physical fatigue, too. This is possibly caused by tyrosine kinase inhibitors (TKIs) used for therapy of renal cell carcinoma and non‐small cell lung cancer as fatigue has been shown to be a major side effect of several TKIs.^22^, ^23^, ^24^

Furthermore, after adjusting for the different treatment modalities and patient characteristics, physical fatigue levels were significantly higher among endometrial compared to breast cancer patients. Although the difference failed statistical significance after adjusting for multiple testing (*P* = .17), it might be worth considering that fatigue in endometrial cancer has been found to be associated with menopausal symptoms, which are a common consequence of the surgical procedures for this type of cancer that result in estrogen deficiency.^25^ Thus, hormonal pathways may be more relevant in fatigue etiology with this type of cancer than in many other cancer types.

Surprisingly, bladder cancer patients had the highest prevalence of emotional fatigue (49%) although prevalence for physical fatigue (38%) was among the lowest of all considered entities. The adjusted mean emotional fatigue was significantly higher than among breast cancer patients. A recent review found that bladder cancer often suffer from depression and anxiety,^26^ possibly because social participation and emotional well‐being are impacted by incontinence, frequent and painful urination, embarrassment, and fear of catheter insertion or removal.^27^ Similar problems might contribute to the high adjusted mean emotional fatigue among rectal cancer patients who often suffer from stool or urinary incontinence and partly may need a stoma, which can impact mental health.^28^, ^29^

Overall, all potential causes or contributing factors for fatigue may need more attention regarding the management of fatigue. Up‐to‐date, recommendations usually do not differentiate by entity or individual patients' and treatment characteristics. Likewise, intervention studies typically follow a one‐fits‐all approach. However, fatigue management and treatment may need to be more individualized, for example, taking entity‐specific problems, cancer therapy, nutritional status, physical condition, and psycho‐social factors into account. Additionally, our results showing that fatigue is prevalent even 2 years after diagnosis across all investigated entities underlines the need to integrate the recommendations for a systematic fatigue management into long‐term aftercare.

Limitations and strengths of our study need to be considered. We cannot exclude a selection bias due to the limited response rate, which could result in (1) underestimation, as patients with fatigue might have been too exhausted to participate, as well as in (2) overestimation as patients without fatigue might not have been interested to participate, because they were not affected by this problem. However, low response was also caused by the formal two‐step procedure required by legislation for data protection issues. The first step, that is, asking by postal mail for patient's consent to transfer his or her contact data to the study center, was a major hurdle: 31% of contacted patients did not agree. Furthermore, from 36% of contacted patients—especially with cancer entities that tend to have worse prognosis—the cancer registry received no response at all. It can be speculated that one major reason for nonresponse may have been poor health status or being already in a palliative situation. However, as advanced tumor stage, the presence of metastases, and a poorer performance status have been shown previously to be associated with higher fatigue levels,^30^ our study finding of differential fatigue across cancer entities, with low fatigue among breast cancer and highest fatigue among stomach cancer, might be rather conservative than biased into a false direction. This was supported by sensitivity analyses estimating the “true” fatigue prevalence under different scenarios, for example, assuming a high fatigue prevalence of 60% among non‐participants. Overall, although bias due to low response rate cannot be excluded, we believe that the study data still yields reliable and valuable results. Yet, for lung, pancreas, and liver cancer the results should be interpreted with caution due to low sample size. Furthermore, we had only limited data on metastases. Thus, we could not stratify fatigue prevalence by disease stage. Moreover we got feedback from several contacted persons or their relatives that participation was declined due to poor physical or mental health, for example, dementia. Patients with insufficient German language skills also could not participate. Cognitive fatigue might be underestimated, since patients with severe cognitive fatigue may have been less willing or able to participate. Moreover to limit the length of the survey, we did not collect information on other potential confounders such as physical activity or social support. Strengths of the study include the systematic and comparable assessment of fatigue across a variety of common cancer entities (albeit with limited sample size for some entities), the systematic, representative sampling via a population‐based statewide cancer registry, the relatively high sample size, and the consideration of different fatigue dimensions.

## 5 CONCLUSIONS

Our study among cancer patients showed that physical, emotional, and cognitive fatigue is prevalent in all 15 investigated entities even approximately 2 years after diagnosis. Yet, there are differences between entities, which are not solely attributable to differences in sex, age, BMI, and cancer therapies. For most cancer types, fatigue levels were above those of breast cancer patients—the latter being the group investigated most in respect to fatigue so far. Thus, the extent of this burdensome problem is probably still insufficiently determined and recognized.

## CONFLICT OF INTEREST

None.

## AUTHOR CONTRIBUTIONS

Martina Schmidt contributed to conceptualization, supervision, formal analysis, and writing—original draft. Silke Hermann contributed to resources and writing—review and editing. Volker Arndt contributed to resources and writing—review and editing. Karen Steindorf contributed to conceptualization and writing—review and editing.

## COMPLIANCE WITH ETHICAL STANDARDS

This study was conducted in accordance with the ethical standards of the Helsinki Declaration. The study was approved by the Ethic Committee of the Medical Faculty of the University of Heidelberg. All patients have given written informed consent.

## Supporting information

Table S1

Table S2

Table S3

Table S4

## Data Availability

Data available on request due to privacy/ethical restrictions.
