# Supplementary material for: Prevalence and severity of long‐term physical, emotional, and cognitive fatigue across 15 different cancer entities
Source: Cancer Med. 2020 Sep 7;9(21):8053–61. doi: 10.1002/cam4.3413 (PMC7643651; doi:10.1002/cam4.3413)
Supplement: Supplementary file 1 — Table S1 [file CAM4-9-8053-s001.docx]

**Table S1:** Physical fatigue by entity, sex and age

|  | **Male** | | | | | | | | **Female** | | | | | | | |
| --- | --- | --- | --- | --- | --- | --- | --- | --- | --- | --- | --- | --- | --- | --- | --- | --- |
|  | **< 65 years** | | | | **≥ 65 years** | | | | **< 65 years** | | | | **≥ 65 years** | | | |
| **Entity** | **N** | **Median** | **Q1** | **Q3** | **N** | **Median** | **Q1** | **Q3** | **N** | **Median** | **Q1** | **Q3** | **N** | **Median** | **Q1** | **Q3** |
| Bladder | 33 | 33.3 | 13.3 | 66.7 | 79 | 33.3 | 16.7 | 66.7 | 8 | 20.0 | 0.0 | 53.3 | 18 | 33.3 | 6.7 | 46.7 |
| Breast | 0 |  |  |  | 6 | 43.3 | 33.3 | 66.7 | 158 | 33.3 | 13.3 | 60.0 | 64 | 41.7 | 20.0 | 66.7 |
| Colon | 42 | 33.3 | 13.3 | 53.3 | 73 | 40.0 | 20.0 | 60.0 | 24 | 33.3 | 10.0 | 66.7 | 43 | 46.7 | 26.7 | 80.0 |
| Endometrium | n.a. |  |  |  |  | n.a. |  |  | 79 | 46.7 | 26.7 | 66.7 | 93 | 40.0 | 20.0 | 66.7 |
| Kidney | 67 | 33.3 | 20.0 | 53.3 | 75 | 33.3 | 13.3 | 60.0 | 33 | 60.0 | 46.7 | 73.3 | 30 | 40.0 | 20.0 | 66.7 |
| Leukemia | 46 | 33.3 | 20.0 | 66.7 | 42 | 26.7 | 13.3 | 46.7 | 33 | 53.3 | 20.0 | 73.3 | 37 | 40.0 | 26.7 | 66.7 |
| Liver | 6 | 26.7 | 13.3 | 60.0 | 11 | 50.0 | 6.7 | 60.0 | 5 | 33.3 | 26.7 | 73.3 | 7 | 53.3 | 0.0 | 86.7 |
| Lung | 10 | 33.3 | 25.0 | 66.7 | 12 | 43.3 | 26.7 | 60.0 | 7 | 73.3 | 26.7 | 80.0 | 6 | 70.0 | 40.0 | 80.0 |
| Malignant melanoma | 32 | 36.7 | 6.7 | 60.0 | 46 | 30.0 | 13.3 | 46.7 | 54 | 46.7 | 26.7 | 66.7 | 30 | 33.3 | 20.0 | 60.0 |
| Non-Hodgkin lymphoma | 57 | 40.0 | 20.0 | 66.7 | 58 | 46.7 | 26.7 | 66.7 | 41 | 40.0 | 20.0 | 60.0 | 47 | 46.7 | 13.3 | 60.0 |
| Ovaries | n.a. |  |  |  | n.a. |  |  |  | 95 | 46.7 | 26.7 | 66.7 | 50 | 40.0 | 20.0 | 66.7 |
| Pancreas | 9 | 40.0 | 33.3 | 73.3 | 8 | 33.3 | 13.3 | 66.7 | 6 | 46.7 | 26.7 | 66.7 | 10 | 66.7 | 33.3 | 93.3 |
| Prostate | 58 | 26.7 | 13.3 | 53.3 | 159 | 26.7 | 13.3 | 53.3 | n.a. |  |  |  | n.a. |  |  |  |
| Rectum | 51 | 40.0 | 20.0 | 60.0 | 68 | 33.3 | 13.3 | 46.7 | 33 | 53.3 | 20.0 | 73.3 | 38 | 43.3 | 13.3 | 73.3 |
| Stomach | 35 | 46.7 | 20.0 | 66.7 | 42 | 53.3 | 33.3 | 80.0 | 16 | 50.0 | 40.0 | 66.7 | 29 | 40.0 | 26.7 | 60.0 |
