# Supplementary material for: Prevalence and severity of long‐term physical, emotional, and cognitive fatigue across 15 different cancer entities
Source: Cancer Med. 2020 Sep 7;9(21):8053–61. doi: 10.1002/cam4.3413 (PMC7643651; doi:10.1002/cam4.3413)
Supplement: Supplementary file 2 — Table S2 [file CAM4-9-8053-s002.docx]

**Table S2:** Emotional fatigue by entity, sex and age

|  | **Male** | | | | | | | | **Female** | | | | | | | |
| --- | --- | --- | --- | --- | --- | --- | --- | --- | --- | --- | --- | --- | --- | --- | --- | --- |
|  | **< 65 years** | | | | **≥ 65 years** | | | | **< 65 years** | | | | **≥ 65 years** | | | |
| **Entity** | **N** | **Median** | **Q1** | **Q3** | **N** | **Median** | **Q1** | **Q3** | **N** | **Median** | **Q1** | **Q3** | **N** | **Median** | **Q1** | **Q3** |
| Bladder | 33 | 11.1 | 0.0 | 33.3 | 79 | 11.1 | 0.0 | 33.3 | 8 | 22.2 | 0.0 | 61.1 | 18 | 33.3 | 0.0 | 33.3 |
| Breast | 0 |  |  |  | 6 | 22.2 | 11.1 | 55.6 | 158 | 11.1 | 0.0 | 33.3 | 65 | 16.7 | 0.0 | 33.3 |
| Colon | 42 | 0.0 | 0.0 | 33.3 | 73 | 0.0 | 0.0 | 22.2 | 24 | 11.1 | 0.0 | 38.9 | 43 | 22.2 | 0.0 | 44.4 |
| Endometrium | n.a. |  |  |  | n.a. |  |  |  | 79 | 11.1 | 0.0 | 44.4 | 92 | 16.7 | 0.0 | 44.4 |
| Kidney | 67 | 11.1 | 0.0 | 33.3 | 75 | 0.0 | 0.0 | 44.4 | 33 | 33.3 | 11.1 | 66.7 | 30 | 5.6 | 0.0 | 22.2 |
| Leukemia | 46 | 11.1 | 0.0 | 33.3 | 42 | 0.0 | 0.0 | 22.2 | 33 | 22.2 | 11.1 | 55.6 | 37 | 11.1 | 0.0 | 33.3 |
| Liver | 6 | 5.6 | 0.0 | 11.1 | 11 | 11.1 | 0.0 | 33.3 | 5 | 11.1 | 0.0 | 33.3 | 7 | 22.2 | 0.0 | 44.4 |
| Lung | 10 | 5.6 | 0.0 | 33.3 | 12 | 11.1 | 11.1 | 22.2 | 7 | 22.2 | 11.1 | 66.7 | 6 | 44.4 | 11.1 | 55.6 |
| Malignant melanoma | 32 | 11.1 | 0.0 | 27.8 | 46 | 0.0 | 0.0 | 33.3 | 54 | 11.1 | 0.0 | 33.3 | 30 | 22.2 | 0.0 | 33.3 |
| Non-Hodgkin lymphoma | 57 | 22.2 | 0.0 | 33.3 | 58 | 11.1 | 0.0 | 33.3 | 41 | 11.1 | 0.0 | 33.3 | 47 | 11.1 | 0.0 | 33.3 |
| Ovaries | n.a. |  |  |  | n.a. |  |  |  | 95 | 11.1 | 0.0 | 44.4 | 50 | 11.1 | 0.0 | 44.4 |
| Pancreas | 9 | 0.0 | 0.0 | 33.3 | 8 | 16.7 | 0.0 | 38.9 | 6 | 27.8 | 11.1 | 44.4 | 10 | 44.4 | 11.1 | 55.6 |
| Prostate | 58 | 0.0 | 0.0 | 33.3 | 161 | 0.0 | 0.0 | 22.2 | n.a. |  |  |  | n.a. |  |  |  |
| Rectum | 51 | 22.2 | 0.0 | 44.4 | 68 | 0.0 | 0.0 | 33.3 | 33 | 33.3 | 0.0 | 66.7 | 38 | 27.8 | 0.0 | 66.7 |
| Stomach | 35 | 22.2 | 0.0 | 44.4 | 42 | 16.7 | 0.0 | 44.4 | 16 | 16.7 | 5.6 | 38.9 | 28 | 11.1 | 0.0 | 38.9 |
