# Supplementary material for: Prevalence and severity of long‐term physical, emotional, and cognitive fatigue across 15 different cancer entities
Source: Cancer Med. 2020 Sep 7;9(21):8053–61. doi: 10.1002/cam4.3413 (PMC7643651; doi:10.1002/cam4.3413)
Supplement: Supplementary file 3 — Table S3 [file CAM4-9-8053-s003.docx]

**Table S3:** Total fatigue by entity, sex and age

|  | **Male** | | | | | | | | **Female** | | | | | | | |
| --- | --- | --- | --- | --- | --- | --- | --- | --- | --- | --- | --- | --- | --- | --- | --- | --- |
|  | **< 65 years** | | | | **≥ 65 years** | | | | **< 65 years** | | | | **≥ 65 years** | | | |
| **Entity** | **N** | **Median** | **Q1** | **Q3** | **N** | **Median** | **Q1** | **Q3** | **N** | **Median** | **Q1** | **Q3** | **N** | **Median** | **Q1** | **Q3** |
| Bladder | 33 | 22.2 | 8.3 | 41.7 | 79 | 27.8 | 8.3 | 47.2 | 8 | 16.8 | 1.4 | 52.8 | 18 | 26.8 | 2.8 | 36.1 |
| Breast | 0 |  |  |  | 6 | 30.6 | 19.4 | 47.2 | 158 | 25.0 | 8.3 | 44.4 | 65 | 30.6 | 11.1 | 50.0 |
| Colon | 42 | 22.2 | 8.3 | 38.9 | 73 | 22.2 | 11.1 | 36.1 | 24 | 20.8 | 6.9 | 47.2 | 43 | 36.1 | 16.7 | 50.0 |
| Endometrium | n.a. |  |  |  | n.a. |  |  |  | 79 | 27.8 | 13.9 | 50.0 | 93 | 27.8 | 13.9 | 47.2 |
| Kidney | 67 | 19.4 | 11.1 | 38.9 | 75 | 19.4 | 8.3 | 41.7 | 33 | 47.2 | 27.3 | 61.1 | 30 | 25.0 | 8.3 | 41.7 |
| Leukemia | 46 | 25.0 | 11.1 | 50.0 | 42 | 15.3 | 5.6 | 30.3 | 33 | 36.1 | 11.1 | 52.8 | 37 | 30.6 | 13.9 | 41.7 |
| Liver | 6 | 15.3 | 5.6 | 36.1 | 11 | 30.6 | 3.0 | 41.7 | 5 | 19.4 | 13.9 | 52.8 | 7 | 30.6 | 0.0 | 55.6 |
| Lung | 10 | 24.4 | 11.1 | 36.1 | 12 | 23.6 | 18.1 | 31.9 | 7 | 38.9 | 19.4 | 75.0 | 6 | 54.2 | 22.2 | 55.6 |
| Malignant melanoma | 32 | 22.2 | 2.8 | 34.7 | 46 | 16.7 | 5.6 | 36.1 | 54 | 33.3 | 13.9 | 44.4 | 30 | 20.8 | 8.3 | 41.7 |
| Non-Hodgkin lymphoma | 57 | 30.6 | 15.2 | 44.4 | 58 | 27.8 | 13.9 | 44.4 | 41 | 25.0 | 11.1 | 44.4 | 47 | 25.0 | 5.6 | 44.4 |
| Ovaries | n.a. |  |  |  | n.a. |  |  |  | 95 | 30.6 | 13.9 | 52.8 | 50 | 25.0 | 16.7 | 47.2 |
| Pancreas | 9 | 27.8 | 16.7 | 44.4 | 8 | 27.8 | 5.6 | 43.1 | 6 | 31.9 | 16.7 | 55.6 | 10 | 45.8 | 22.2 | 63.9 |
| Prostate | 58 | 19.4 | 5.6 | 38.9 | 161 | 13.9 | 5.6 | 36.1 | n.a. |  |  |  | n.a. |  |  |  |
| Rectum | 51 | 25.0 | 13.9 | 47.2 | 68 | 18.1 | 5.8 | 34.7 | 33 | 33.3 | 8.3 | 61.1 | 38 | 33.3 | 8.3 | 63.9 |
| Stomach | 35 | 27.8 | 9.1 | 50.0 | 42 | 33.3 | 18.2 | 58.3 | 16 | 36.1 | 25.0 | 51.4 | 29 | 22.2 | 13.9 | 44.4 |
