# Supplementary material for: Prevalence and severity of long‐term physical, emotional, and cognitive fatigue across 15 different cancer entities
Source: Cancer Med. 2020 Sep 7;9(21):8053–61. doi: 10.1002/cam4.3413 (PMC7643651; doi:10.1002/cam4.3413)
Supplement: Supplementary file 4 — Table S4 [file CAM4-9-8053-s004.docx]

**Table S4:** Mean fatigue values and differences from age- and sex-matched normative values

|  | **Physical fatigue** | | | | |  | **Emotional fatigue** | | | | | |  | | **Cognitive fatigue** | | | | | | | | | | |
| --- | --- | --- | --- | --- | --- | --- | --- | --- | --- | --- | --- | --- | --- | --- | --- | --- | --- | --- | --- | --- | --- | --- | --- | --- | --- |
| **Entity** | **N** | **Mean** | **SD** | **Mean Diff (95%CI)^a^** | **p^b^** |  | **N** | **Mean** | **SD** | **Mean Diff (95%CI)^a^** | | **p^b^** | |  | | **N** | | **Mean** | | **SD** | | **Mean Diff (95%CI)^a^** | | **p^b^** |  |
| Bladder | 139 | 38.3 | 29.1 | 14.5 (9.6, 19.4) | <.0001 |  | 139 | 23.3 | 26.6 | 12.6 (8.2, 17.1) | <.0001 | |  | | 135 | | 11.4 | | 17.3 | | 4.8 (1.8, 7.7) | | 0.0143 | | |
| Breast | 228 | 41.7 | 28.6 | 13.5 (9.8, 17.3) | <.0001 |  | 229 | 22.5 | 26.5 | 7.7 (4.2, 11.2) | 0.0001 | |  | | 224 | | 12.6 | | 20.4 | | 4.3 (1.6, 7.0) | | 0.0143 | | |
| Colon | 182 | 41.7 | 28.3 | 16.9 (12.8, 21.0) | <.0001 |  | 182 | 19.9 | 26.2 | 8.3 (4.5, 12.1) | 0.0002 | |  | | 180 | | 14.1 | | 21.4 | | 7.1 (3.9, 10.2) | | 0.0002 | | |
| Endometrium | 172 | 45.6 | 27.5 | 17.4 (13.3, 21.6) | <.0001 |  | 171 | 25.8 | 28 | 11.6 (7.4, 15.8) | <.0001 | |  | | 170 | | 13 | | 19.6 | | 5.3 (2.3, 8.2) | | 0.0054 | | |
| Kidney | 205 | 42.4 | 27.3 | 18.1 (14.4, 21.8) | <.0001 |  | 205 | 21.2 | 26.2 | 9.6 (6.1, 13.2) | <.0001 | |  | | 202 | | 12.6 | | 21.5 | | 5.5 (2.6, 8.5) | | 0.0028 | | |
| Leukemia | 158 | 40.5 | 28 | 15.6 (11.3, 20.0) | <.0001 |  | 158 | 21.3 | 26.1 | 9.2 (5.2, 13.3) | <.0001 | |  | | 156 | | 10.9 | | 17.9 | | 3.6 (0.8, 6.4) | | 0.0589 | | |
| Liver | 29 | 41.6 | 30.9 | 16.4 (4.8, 28.0) | 0.0074 |  | 29 | 17.6 | 21.9 | 5.9 (-2.5, 14.3) | 0.1645 | |  | | 28 | | 12.5 | | 17.3 | | 5.5 (-1.3, 12.3) | | 0.183 | | |
| Lung | 35 | 48.6 | 27.3 | 24.0 (14.8, 33.2) | <.0001 |  | 35 | 27 | 27.7 | 15.5 (6.1, 24.9) | 0.0057 | |  | | 35 | | 14 | | 21.8 | | 7.1 (-0.3, 14.6) | | 0.183 | | |
| Malignant melanoma | 163 | 38.1 | 27 | 12.5 (8.3, 16.7) | <.0001 |  | 163 | 21 | 26.2 | 8.4 (4.3, 12.4) | 0.0003 | |  | | 162 | | 10.3 | | 20.4 | | 2.8 (-0.4, 5.9) | | 0.183 | | |
| Non-Hodgkin lymphoma | 203 | 42.8 | 28.2 | 18.1 (14.1, 22.0) | <.0001 |  | 203 | 20.9 | 23.6 | 9.0 (5.7, 12.3) | <.0001 | |  | | 200 | | 12.3 | | 18.7 | | 5.1 (2.4, 7.7) | | 0.0022 | | |
| Ovaries | 145 | 45.1 | 26.8 | 16.9 (12.5, 21.3) | <.0001 |  | 145 | 26.2 | 28.6 | 11.5 (6.8, 16.2) | <.0001 | |  | | 144 | | 14.9 | | 20.9 | | 6.6 (3.1, 10.0) | | 0.0025 | | |
| Pancreas | 33 | 49.5 | 31 | 24.3 (13.3, 35.3) | 0.0002 |  | 33 | 27.1 | 27.1 | 15.2 (5.6, 24.7) | 0.0057 | |  | | 33 | | 15.7 | | 18.6 | | 8.6 (2.0, 15.2) | | 0.0589 | | |
| Prostate | 217 | 32.9 | 27.1 | 9.7 (6.0, 13.3) | <.0001 |  | 219 | 16.4 | 25.3 | 6.1 (2.8, 9.5) | 0.0016 | |  | | 216 | | 10.5 | | 20.6 | | 3.9 (1.2, 6.7) | | 0.0317 | | |
| Rectum | 190 | 41 | 28.6 | 16.3 (12.3, 20.4) | <.0001 |  | 190 | 26.2 | 29.9 | 14.4 (10.1, 18.6) | <.0001 | |  | | 189 | | 14.7 | | 21.7 | | 7.6 (4.5, 10.7) | | <.0001 | | |
| Stomach | 123 | 47.7 | 29 | 23.3 (18.1, 28.5) | <.0001 |  | 122 | 25.5 | 28.4 | 14.0 (8.9, 19.1) | <.0001 | |  | | 122 | | 17.8 | | 24.4 | | 10.8 (6.4, 15.1) | | <.0001 | | |

a Mean of individual differences from age- and sex-matched normative values

b p-values of paired t-tests adjusted using the Bonferroni-Holm method
